# Novel Cell-Free Strategy for Therapeutic Angiogenesis: *In Vitro* Generated Conditioned Medium Can Replace Progenitor Cell Transplantation

**DOI:** 10.1371/journal.pone.0005643

**Published:** 2009-05-21

**Authors:** Stefano Di Santo, Zijiang Yang, Moritz Wyler von Ballmoos, Jan Voelzmann, Nicolas Diehm, Iris Baumgartner, Christoph Kalka

**Affiliations:** Department of Vascular Medicine, Swiss Cardiovascular Center, Inselspital, Bern University Hospital, University of Bern, Bern, Switzerland; City of Hope Medical Center, United States of America

## Abstract

**Background:**

Current evidence suggests that endothelial progenitor cells (EPC) contribute to ischemic tissue repair by both secretion of paracrine factors and incorporation into developing vessels. We tested the hypothesis that cell-free administration of paracrine factors secreted by cultured EPC may achieve an angiogenic effect equivalent to cell therapy.

**Methodology/Principal Findings:**

EPC-derived conditioned medium (EPC-CM) was obtained from culture expanded EPC subjected to 72 hours of hypoxia. *In vitro*, EPC-CM significantly inhibited apoptosis of mature endothelial cells and promoted angiogenesis in a rat aortic ring assay. The therapeutic potential of EPC-CM as compared to EPC transplantation was evaluated in a rat model of chronic hindlimb ischemia. Serial intramuscular injections of EPC-CM and EPC both significantly increased hindlimb blood flow assessed by laser Doppler (81.2±2.9% and 83.7±3.0% vs. 53.5±2.4% of normal, P<0.01) and improved muscle performance. A significantly increased capillary density (1.62±0.03 and 1.68±0.05/muscle fiber, P<0.05), enhanced vascular maturation (8.6±0.3 and 8.1±0.4/HPF, P<0.05) and muscle viability corroborated the findings of improved hindlimb perfusion and muscle function. Furthermore, EPC-CM transplantation stimulated the mobilization of bone marrow (BM)-derived EPC compared to control (678.7±44.1 vs. 340.0±29.1 CD34^+^/CD45^−^ cells/1×10^5^ mononuclear cells, P<0.05) and their recruitment to the ischemic muscles (5.9±0.7 vs. 2.6±0.4 CD34^+^ cells/HPF, P<0.001) 3 days after the last injection.

**Conclusions/Significance:**

Intramuscular injection of EPC-CM is as effective as cell transplantation for promoting tissue revascularization and functional recovery. Owing to the technical and practical limitations of cell therapy, cell free conditioned media may represent a potent alternative for therapeutic angiogenesis in ischemic cardiovascular diseases.

## Introduction

Cell-based revascularization therapies have recently been tested in clinical trials investigating the therapeutic benefits in patients that suffer from ischemic cardiovascular diseases [1], [2]. Most of these studies used autologous cell transplantation given the concern of immune-system reactions. Distinct progenitor and stem cell lines have been described for their outstanding potential to promote tissue revascularization and functional recovery of the affected organ. Thus, a variety of progenitor and stem cell types, isolated from bone marrow and peripheral blood, have been used in patients with myocardial infarction, heart failure and peripheral vascular disease [3]. However, technical and practical limitations due to the invasive methods of harvest and low abundance may hinder the adoption of progenitor cells in clinical applications.

Two predominant mechanisms by which progenitor cells like endothelial progenitor cells (EPC) contribute to postnatal neovascularization have been identified so far [4]–[7]. *In vivo* animal studies demonstrated that EPC contribute to vessel formation by differentiation into mature endothelial cells and incorporation into the growing vessel wall [8], [9]. However, this mechanism seems to play only a marginal role [10]–[13]. Secondly, circulating EPC isolated from peripheral blood have been shown to release a number of proangiogenic factors [5], [14]. As a matter of fact, conditioned medium obtained from EPC cultures contains various proangiogenic growth factors and may therefore support the repair and re-endothelization of injured vessels and thus the regeneration of ischemic tissues [15]–[17]. We hypothesized that the regenerative potential of paracrine factors secreted by EPC may represent a potent alternative to progenitor cell therapy.

## Results

### Secretion of growth factors by EPC is increased by hypoxia

We have measured the release of different growth factors to determine to what extent hypoxia enhances the paracrine activity of EPC. Indeed, hypoxia (1.5% O_2_) induced a significant increase in accumulation of selected factors like Angiogenin, HGF, IL-8, PDGF-BB, SDF-1 and VEGF-A in the EPC conditioned media compared to normoxia (P<0.05; Table 1). This effect was due to an augmented secretion since the overall EPC number was not significantly influenced by the oxygen level during culture (data not shown). Therefore, the growth factor enriched EPC-CM from hypoxic cultures was used for subsequent experiments.

**Table 1:** Concentration of selected angiogenic growth factors in EPC-CM.

Cytokine/Growth factor	Concentration (pg/ml)	
	Hypoxia	Normoxia
IL-8/CXCL8	29090.7±12279.4	2282.1±406.3
SDF-1/CXCL12	6059.9±654.6	3179.9±488.0
HGF	539.5±141.7	343.4±74.8
Angiogenin	144.6±68.2	72.5±15.8
PDGF-BB	111.6±27.02	19.9±2.2
VEGF-A	25.5±4.8	11.4±5.2

Selected cytokine levels were measured in the conditioned media from culture expanded EPC incubated in hypoxic or normoxic condition for 72 hours.

### EPC-CM enhances endothelial cell-viability *in vitro*

**Figure 1:**
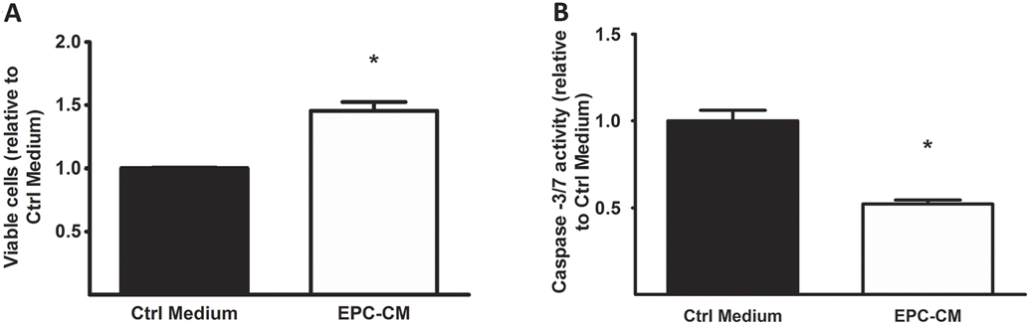
Pro-survival properties of EPC-CM. Serum starved HUVEC were incubated in EPC-CM or control medium for 24 hrs and analyzed for cell survival and extent of apoptosis. (A) The number of viable cells was assessed by CyQuant® NF and expressed relative to control. (B) Apoptosis was measured by the level of caspase −3/7 activity by Apo-ONE® and expressed relative to control. *, P<0.001.

The capacity of EPC-CM to support the viability of nutrient depleted HUVEC was assessed by an assay for survival and for apoptosis. Incubation of EPC-CM resulted in a 45.4±7.0% increase of viable cells compared to control medium (P<0.001; Figure 1A). In contrast, the caspase −3/7 activity was reduced to 52.3±2.3% in HUVEC incubated with EPC-CM compared to control medium (P<0.001; Figure 1B).

### EPC-CM increases vascular sprouting

**Figure 2:**
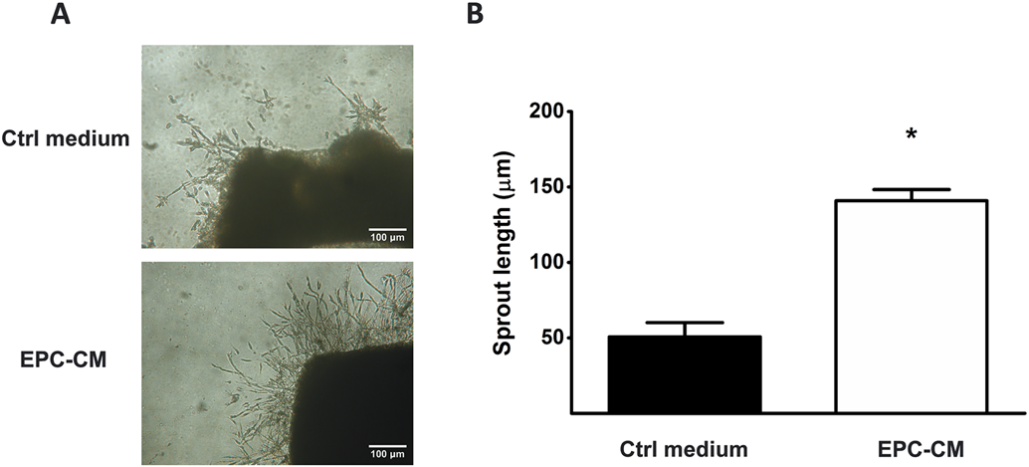
Angiogenic potential of EPC-CM. (A) Representative pictures of vascular outgrowth from 1 mm rat aortic ring embedded in growth factor reduced-Matrigel™ and incubated with EPC-CM or control medium. Incubation with EPC-CM enhanced the formation of capillary outgrowth compared to control medium. (B) Quantitative analysis of sprout length induced by incubation with control medium and EPC-CM. *, P<0.001.

The *ex vivo* aortic ring assay is commonly used to study the outgrowth of endothelial and surrounding perivascular cells and their organization in tubular, vessel-like structures. EPC-CM showed a substantially higher angiogenic potential to stimulate vessel outgrowth from the aortic ring in comparison to control medium. This was evidenced by a significantly wider (50.69±9.41 vs. 140.90±7.41 µm, P<0.001) and clearly denser network of vascular sprouts arising from the aorta (Figure 2).

### EPC-CM and EPC transplantation both improve hindlimb perfusion and muscle function

**Figure 3:**
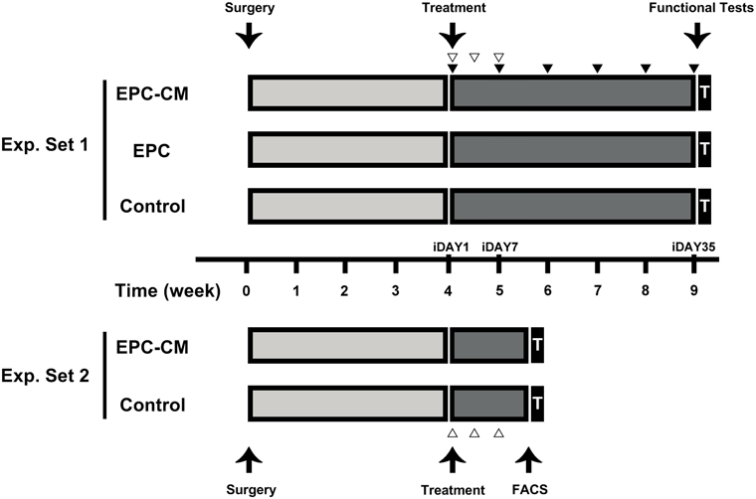
Design of *in vivo* experiments. Two *in vivo* experimental settings were designed to address the effect of the treatment modalities on tissue regeneration and neovascularization (Exp. Set 1) as well as progenitor cells mobilization and recruitment (Exp. Set 2). In both settings, rats were treated by 3 separate intramuscular injections within 7 days (iDAY1- iDAY7), 4 weeks after inducing ischemia as indicated by the white arrowheads (∇). Black arrowheads (▾) indicate blood flow measurements by Laser-Doppler of the hindlimb. T indicates tissue harvest and immunohistochemistry analysis.

**Figure 4:**
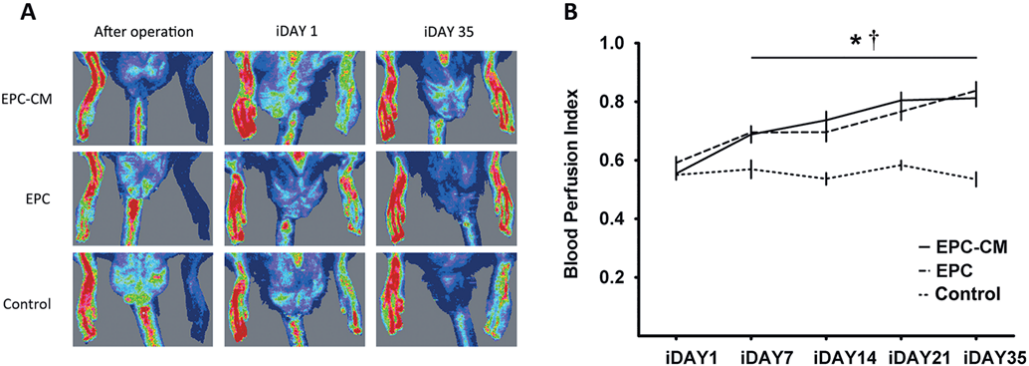
EPC-CM and EPC transplantation improve blood perfusion in the ischemic hindlimb. (A) Representative images of hindlimb blood flow measured by laser Doppler immediately after intramuscular injection of EPC-CM, EPC or control medium (iDAY1, 4 weeks after occlusion of the femoral artery) and the end of the experiment (5 weeks after treatment, iDAY35). (B) Quantitative analysis of blood flow expressed as perfusion ratio of the ischemic to the contralateral (non-operated) hindlimb over the observation period (iDAY1: day of EPC-CM or EPC injection; iDAY7; iDAY14; iDAY21; iDAY28 and iDAY35: 1, 2, 3, 4 and 5 weeks after injection, respectively). *, EPC-CM vs. Control, P<0.01; †, EPC vs. Control, P<0.01.

The animal experimental design is illustrated in Figure 3. In the animals receiving control medium, blood flow remained constant throughout the study around 50% of that measured in the non-ischemic limb (53.5±2.4% at five weeks after treatment). In contrast, the rats treated with EPC-CM or EPC showed a significant improvement in blood flow already by one week after the last injection (P<0.01). Subsequently, blood flow increased gradually to a level of 81.2±2.9% (EPC-CM) and 83.7±3.0% (EPC) after five weeks (P = n.s. between the two treated groups; Figure 4A, B).

**Figure 5:**
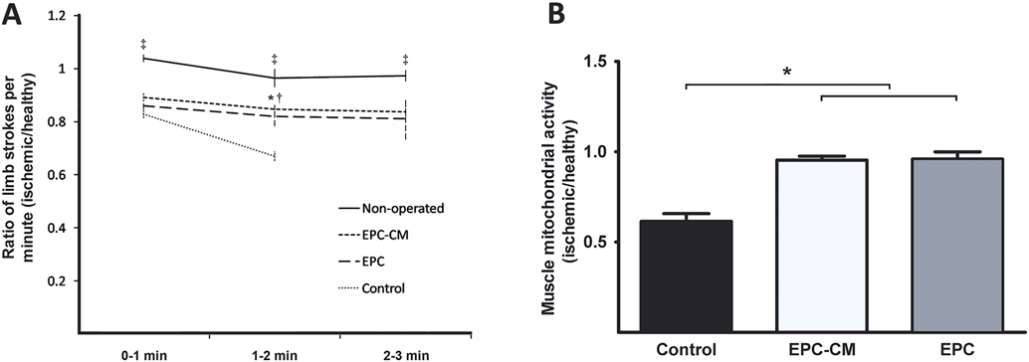
Effect of EPC-CM and EPC transplantation on ischemic muscle function and activity. (A) Muscle function was tested by swimming exercise and expressed as the ratio of ischemic to healthy hindlimb stroke numbers in animals treated with EPC-CM, EPC, control medium or non-operated animals. Swimming activity was monitored for 3 minutes at 1 minute intervals. Rats treated with control medium were not able to complete the exercise due to obvious exhaustion with drowning. *, EPC-CM vs. Control, P<0.05; †, EPC vs. Control, P<0.05; ‡ Non-operated vs. EPC-CM and EPC, P<0.05. (B) Muscle mitochondrial activity in animals treated with EPC-CM, EPC or control medium was assessed by MTT reduction in the healthy and ischemic hindlimbs. The activity index is indicated as the ratio ischemic to healthy MTT values per gram of dry tissue. *, P<0.05.

The improved flow recovery in the ischemic hindlimb was associated with a clear restoration of muscle function. Rats treated with control media had severely limited muscle activity with a stroke ratio decreasing from 0.83±0.02 to 0.67±0.02 within the first two minutes of swimming (P<0.05). The exercise performance deteriorated further until they were unable to swim in the third minute due to obvious exhaustion (near-drowning). In comparison, EPC-CM and EPC treated animals had a significantly better muscle function as evidenced by a stable hindlimb stroke ratio throughout the exercise (EPC-CM, 0–1 min: 0.89±0.02; 1–2 min: 0.85±0.02; 2–3 min: 0.83±0.03 and EPC, 0–1 min: 0.86±0.01; 1–2 min: 0.82±0.04, 2–3 min: 0.81±0.08). The group of non-operated, healthy control animals demonstrated a uniform one to one stroke ratio during the entire exercise protocol (Figure 5A).

Consistent with functional improvement, viability of the ischemic muscle in control medium treated animals was down to 61.4±4.3% of the healthy hindlimb value (Figure 5B), while in the EPC-CM and EPC group the viability was restored to 95.3±2.2% and 95.9±4.0% of healthy muscle (both P<0.05).

### EPC-CM and EPC transplantation equally induce neovascularization and vascular maturation

**Figure 6:**
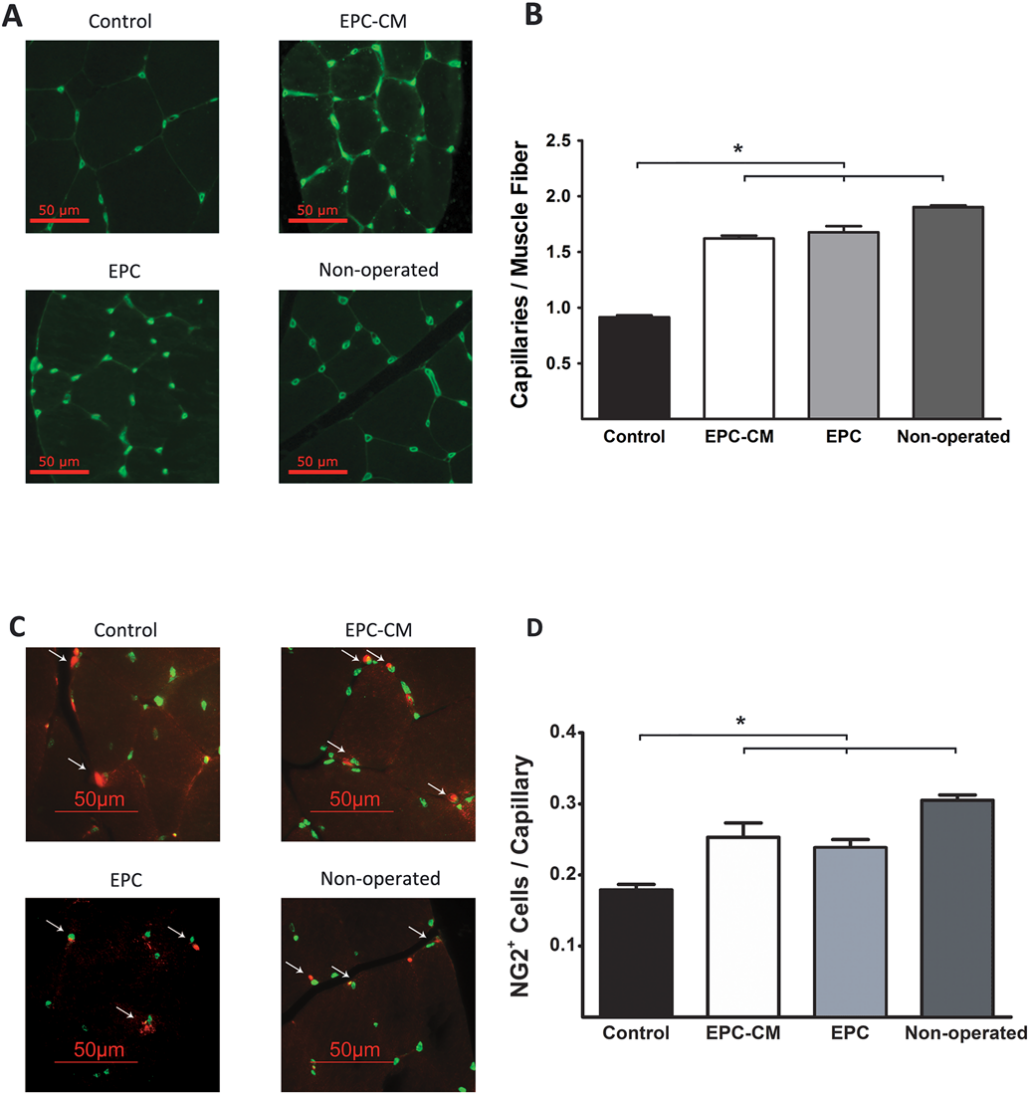
Effect of EPC-CM and EPC transplantation on ischemic muscle neovascularization. (A) Representative images of healthy (non-operated) and ischemic hindlimb muscle of animals treated with EPC-CM, EPC or control medium stained with BS-1 lectin (FITC) to localize capillaries. (B) Quantitative analysis of capillary density expressed by the number of capillaries per muscle fiber. *, P<0.05. (C) NG2^+^ pericytes (white arrows) were identified (red fluorescence) by being adjacent to endothelial cells stained for von Willebrand Factor (green fluorescence). (D) Quantitative analysis of NG2^+^ cells per capillary in healthy and ischemic hindlimbs treated with EPC-CM, EPC and control medium. *, P<0.05.

Five weeks after treatment, the number of capillaries in hindlimbs treated with control media was 0.92±0.02 per muscle fiber which reflects a more than 40% reduction in capillary density in comparison to non-operated, healthy tissue (1.90±0.02, P<0.05). However, treatment with EPC-CM (1.62±0.03, P<0.05) and EPC (1.68±0.05, P<0.05) induced significant increase in capillary density returning the capillary number to almost 90% of that found in a normal, healthy hindlimb (Figure 6A, B). No evidence of focally enhanced vascularization was detected, as the ratio of capillary density/muscle fiber obtained from the gastrocnemius muscle at three different anatomic levels showed a relatively uniform and widespread vascularization (from proximal to distal: 1.67±0.06, 1.64±0.04, 1.62±0.04, P = n.s.).

**Figure 7:**
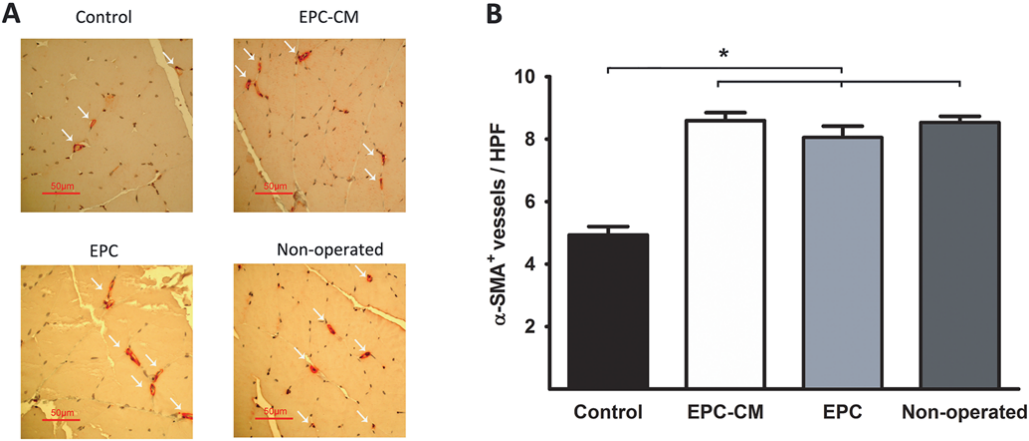
Effect of EPC-CM and EPC transplantation on vascular maturation. (A) Representative images of healthy and ischemic hindlimb muscle of animals treated with EPC-CM, EPC or control medium stained with α-smooth muscle actin (α-SMA) to evidence vascular maturation (red staining, white arrows). (B) Quantitative analysis of α-SMA^+^ vessels per high power field (HPF). *, P<0.05.

Furthermore, the number of NG2^+^ pericytes per capillary was significantly higher in animals treated with EPC-CM (0.25±0.02) or EPC (0.24±0.01) as compared to control medium treated rats (0.18±0.01, P<0.05; Figure 6C, D). In parallel, the number of vessels coated by smooth muscle cells was also significantly higher in animals treated with EPC-CM or EPC (8.6±0.3/HPF and 8.1±0.4/HPF) as compared to controls (4.9±0.3/HPF, P<0.05, Figure 7).

### EPC-CM transplantation stimulates mobilization and recruitment of bone marrow-derived EPC to the ischemic hindlimbs

**Figure 8:**
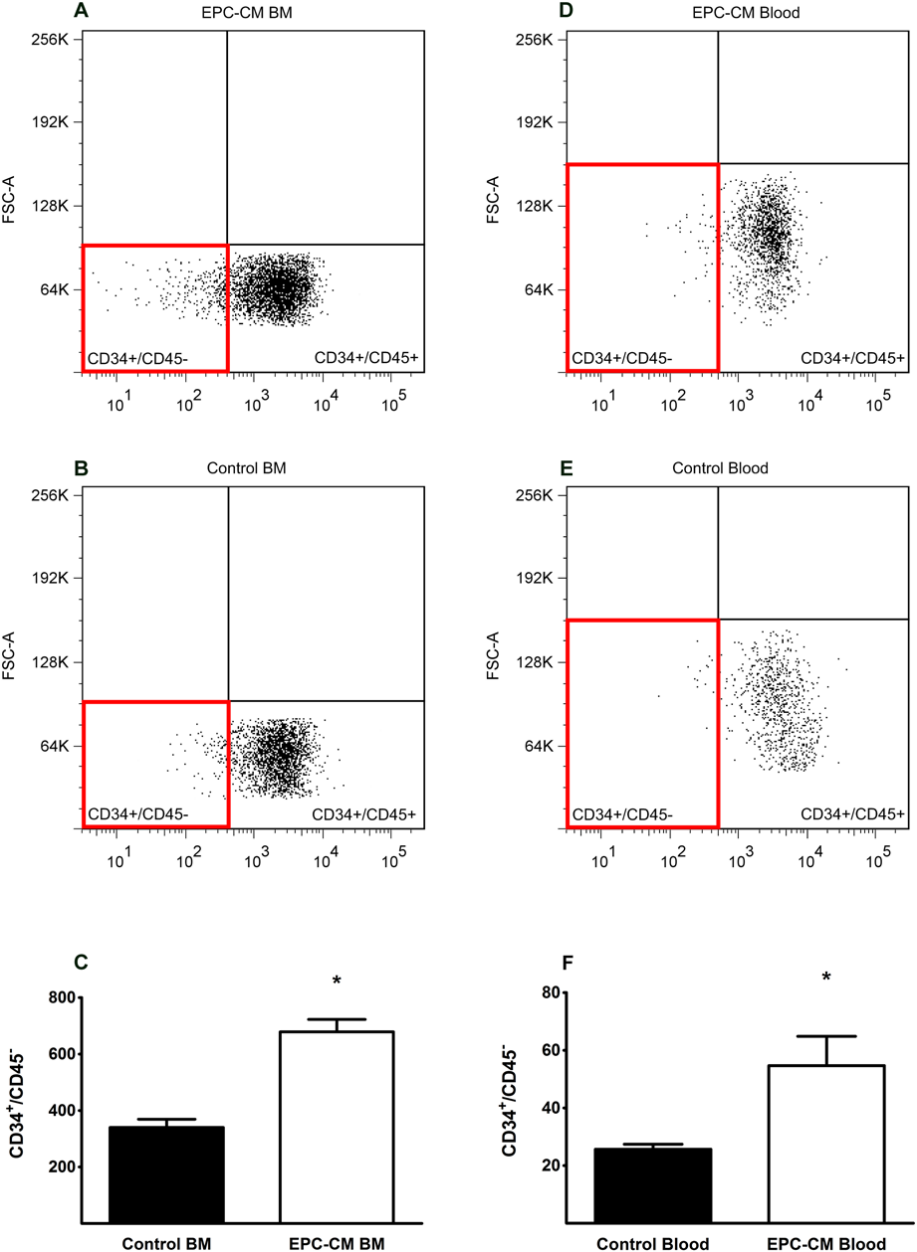
EPC-CM stimulates the mobilization of bone marrow-derived EPC. Representative FACS analysis charts of CD34^+^/CD45^−^ cells isolated from bone marrow (A and B) and peripheral blood (D and E) of EPC-CM and control media treated animals 3 days after the last intramuscular injection. Quantitative analyses show significantly increased numbers of CD34^+^/CD45^−^ progenitor cells in the BM (C), and the peripheral blood (F) of EPC-CM treated animals. *, P<0.05.

**Figure 9:**
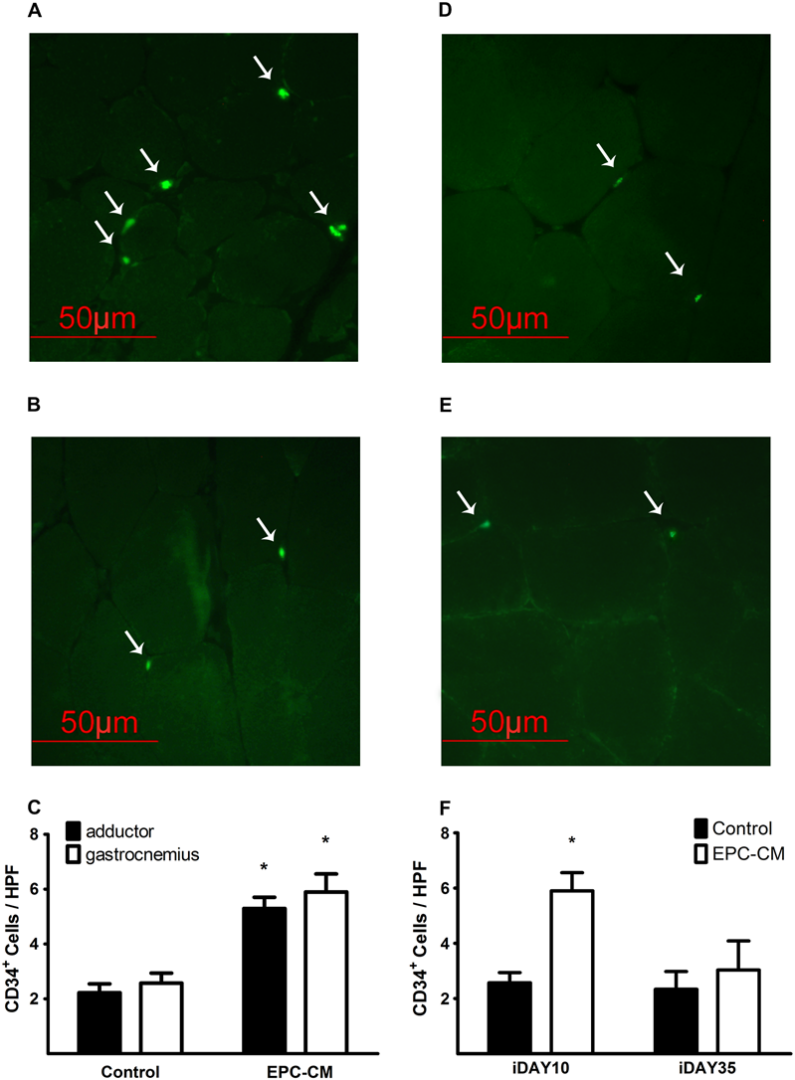
EPC-CM promotes progenitor cells homing to the ischemic tissue. Representative fluoresence pictures of CD34^+^ immunostaining in ischemic hindlimb tissue 3 days (left panel, iDAY10) and 4 weeks after treatment (right panel, iDAY35). The number of CD34^+^ cells on iDAY10 was significantly higher in EPC-CM treated limbs (A) as compared to control treated animals (B) with no evidence for focal recruitment, as CD34^+^ cells were found to similar extent in different anatomic regions (C). In comparison, 4 weeks after treatment (iDAY35), tissue sections from show decreased numbers of CD34^+^ cells in EPC-CM treated limbs (D) equivalent to numbers found in control (E). Quantitative analysis is depicted reflects the temporary recruitment of CD34^+^ cells to the ischemic limbs in EPC-CM treated animals (F). *, P<0.001.

Three days after the last injection, the number of CD34^+^/CD45^−^ progenitor cells/1×10^5^ mononuclear cells (MNC) [18] was significantly elevated in the bone marrow (678.7±44.1 vs. 340.0±29.1, P<0.05, Figure 8A–C) and the peripheral blood (54.7±10.2 vs. 25.7±1.8, P<0.05, Figure 8D–F) of animals treated with EPC-CM as compared to control media. Concomitantly, the number of CD34^+^ cells within the ischemic muscle tissue of EPC-CM treated limbs were significantly higher than in control media treated (5.9±0.7/HPF vs. 2.6±0.4/HPF in gasctrocnemius, P<0.001, Figure 9A, B). Interestingly, numbers of CD34^+^ cells were similar in muscles from different anatomic regions (5.3±0.4/HPF vs. 2.2±0.3/HPF in adductor muscle, P<0.001), suggesting a widespread recruitment of progenitor cells rather than a localized migration (Figure 9C). Analysis of tissue sections from later time points (iDAY 35) showed a decline of CD34^+^ cells to levels similar to control media treated animals (Figure 9D–F). These results indicate a temporary but potent systemic effect of EPC-CM on mobilization and homing of progenitor cells to the ischemic muscle.

## Discussion

In the present study we demonstrate that paracrine factors released by *in vitro* expanded EPC have a potent therapeutic capacity in a rat model of hindlimb ischemia. We present convincing evidence that treatment with EPC-CM leads to a substantial increase in blood flow in the presence of augmented neovascularization, vascular maturation and muscle function in the ischemic hindlimb. Most importantly, the observed regenerative potential after EPC-CM was equivalent to that achieved by EPC transplantation.

Emerging evidence suggests that paracrine signals from stem and progenitor cells are fundamental players in various processes of tissue repair [14], [15], [19], [20] integrating the mechanisms relying on cell differentiation and engraftment. Preclinical studies have described that EPC secretion of factors involved in the regulation of stem cell recruitment and in vascular growth and remodeling (such as SDF-1, VEGF, HGF and MMP-9) [5], [17] support the function of mature endothelial cells *in vitro* and tissue regeneration in a variety of animal models [21], [22]. However, despite the fact that the regenerative capacity of EPC-secreted factors is recognized [23] the spectrum of paracrine effectors and their mechanisms of action remain largely unexplored. Recently the characterization of multifaceted nature of the EPC secretome has been addressed [16] but further investigations are needed to clarify the activation and the interactions of downstream signals.

Although the aim of this work was not the identification of the molecular effectors responsible for the angiogenic properties of EPC-CM, we have confirmed that EPC release key angiogenic molecules such as Angiogenin, HGF, IL-8, PDGF, SDF-1 and VEGF in culture. Consistent with previous studies reporting the stimulatory activity of hypoxia on differentiated endothelial cells and EPC, we found an increased secretory activity under hypoxic conditions [22], [24]. Accordingly, the blend of growth factors contained in the EPC-CM disclosed a strong capacity to sustain fundamental biological functions of mature endothelial cells such as endothelial viability and sprouting. The pro-survival activity of EPC-CM is of utmost relevance in the patho-physiological scenario of chronic muscle tissue ischemia where viability of resident endothelial cells is compromised by the reduced oxygen and nutrient supply [25].

A better understanding of the mechanisms by which cytokines support the functions of resident cells and circulating bone marrow-derived cell populations has led to the development of a number of therapeutic angiogenesis strategies. These include the direct delivery of a variety of recombinant cytokines [26]–[28] or the gene encoding the desired angiogenic protein [29]–[32]. However, clinical trials based on the administration of a single factor have shown contrasting results [31], [33], [34] probably reflecting the fact that the synergic activity of different growth factors is needed to induce formation of stable vascular networks [35], [36]. In contrast to findings after VEGF monotherapy, which promotes intense endothelial sprouting, but results in the development of leaky and disorganized conduits [37], EPC-CM induced the formation of a persistent capillary network as clearly evidenced by long-lasting enhanced density of capillaries and mature vessels as detected five weeks after injection. From this one can speculate that the complex process of revascularization in ischemic tissue is improved by the number of soluble factors present in the EPC-CM, which are presumably able to target simultaneously multiple cell types.

The findings reported here potentially have important implications for the development of novel therapeutic strategies. Most importantly, our data suggest a strategy free from the limitations and problems observed with cell transplantation [38]. It has been described that age and other cardiovascular risk factors reduce the availability and function of EPC, thus limiting their therapeutic applicability in affected patients [39]–[41]. Furthermore, the relative scarcity of circulating EPC and their limited proliferative potential prevent the possibility of expanding these cells in sufficient numbers for some therapeutic applications. Therefore, the use of heterologous cells seems to be the only available option to provide patients suffering from cardiovascular disease with a cell-based therapy. However, immunotolerance concerns and technical as well as practical difficulties may hinder this type of treatment. In contrast, a cell-free medium such as EPC-CM significantly reduces the risk of adverse immunological reactions, simplifies the process of production and thus increases the availability of the therapy. In the present study we have used hypoxic culture conditions to achieve maximum concentrations of growth factors and chemokines in the conditioned medium of EPC [42]. Since the aim of our research was to investigate an alternative therapeutic option to current pre-clinical and clinical EPC transplantation protocols which apply normoxic culture conditions, we have compared the regenerative potential of hypoxic EPC-CM to normoxic EPC cultures. It is of note that the amount of cells required to generate a therapeutic dose of EPC-CM is significantly lower in comparison to the number of EPC needed for transplantation. More precisely, the number of EPC injected was 8-fold higher than the number of cells required to generate the volume of EPC-CM needed to achieve the same therapeutic benefit. We speculate that a considerable number of EPC undergo cell death during and after transplantation. Additionally, the unfavorable microenvironment present in the ischemic tissue might impair the effectiveness of cell transplantation. In contrast, the administration of a mixture of physiologically relevant cytokines and growth factors as by EPC-CM injection, might induce a permissive milieu for differentiated as well as progenitor cells of the host and thus stimulate the endogenous repair system [43]–[45]. The prolonged re-vascularization of the ischemic tissue induced by EPC-CM treatment is intriguing given the short half-life of growth factors. In fact, rapid inactivation and degradation are major limitations of the therapeutic approach using intravenous or intramuscular protein applications [46]. The presented data therefore suggest that in our experimental setting the integrity of the proteins in EPC-CM is preserved resulting in a sustained level and activity of growth factors in the tissue with a long-lasting angiogenic effect.VEGF and SDF-1 may be key factors in amplifying the angiogenic signals of EPC-CM as both have be shown to recruit and entrap pro-angiogenic BM-derived cells in the ischemic tissue [47], [48]. Indeed, our data provide evidence that intramuscular injection of EPC-CM effectively enhanced the number of EPC in the BM, promoted mobilization into the peripheral blood and their homing to the ischemic limbs. In accordance with previous publications, this effect appears to be temporally limited [49]. Interestingly, this recruitment as well as the induction of neovascularization do not show a focal pattern but appear rather equally distributed throughout the ischemic muscle.

Taken together, our observations support the concept that EPC-CM has the potential to replace cell transplantation. Moreover, this study provides a reference for future investigations which will improve our understanding of the regenerative properties of EPC. In particular, knowledge about the differences in healing capacity between EPC-CM obtained from healthy donors and patients with cardiovascular risk factors in combination with the elucidation of their respective secretomes will give the opportunity to define the paracrine functions of EPC in health and disease. These advances will then serve to set up an effective tool to support the defective paracrine processes in the ischemic tissues. It is, therefore, reasonable to imagine that the development of a synthetic preparation which mimics physiological EPC-CM could provide clinicians with a readily available product of standardized quantity and quality.

In conclusion, we have demonstrated in the present study that the constellation of soluble factors secreted by *in vitro* expanded EPC is able to support revascularization of hindlimb ischemic tissue. These data strongly suggest that interventions based on EPC paracrine factors might effectively replace cell transplantation. Future studies designed to identify these factors and the activation of the respective downstream cellular targets might ultimately provide a more effective and practical therapeutic strategy for the treatment of ischemic diseases.

## Materials and Methods

### Cell and conditioned medium preparation

Human peripheral venous blood samples were obtained from healthy, young volunteers (n = 7, age range: 24–38 years) with informed consent. The MNC population was isolated by gradient centrifugation and cultured for 7 days in complete endothelial cell growth medium (EGM-2-MV, Lonza, Switzerland) containing 5% fetal bovine serum (FBS) to obtain EPC in accordance with previously published method [11]. To produce human EPC conditioned medium (EPC-CM), EPC were cultured for 72 hours under hypoxic conditions (1.5% O_2_, 5% CO_2_, 93.5% N_2_) in growth factor-free endothelial cell basal medium-2 (EBM-2, Lonza, Switzerland) with 1% FBS. The conditioned medium was then collected and centrifuged to harvest a cell-free solution. EBM-2 containing 1% FBS without supplements served as control medium. Human umbilical vein Endothelial Cells (HUVEC) were isolated from umbilical cords and cultured using a standard protocol [50]. All protocols received full approval from the Cantonal and the Institutional Ethics Review Board and a signed informed consent was obtained from all participants.

### ELISA and multiplex assay

The concentration of Angiogenin, Hepatocyte Growth Factor (HGF), Interleukin-8 (IL-8), Platelet Derived Growth Factor B (PDGF-BB), Stromal Cell-Derived Factor -1 (SDF-1) and Vascular Endothelial Growth Factor A (VEGF-A) was assessed in EPC culture supernatants generated under normoxic or hypoxic conditions. Angiogenin levels were determined by ELISA (RayBiotech, Norcross GA, USA) whereas the concentration of the other cytokines was done using a multiplex assay (Bioplex, Bio-Rad, Switzerland) following the manufacturer's instructions. All measurements were performed in duplicates from five different donors.

### Cell survival assay

HUVEC were seeded into 96-well plates coated with 1% gelatin and starved in EBM-2 containing 1% FBS for 24 hours. The cells were then exposed to EPC-CM or control medium. After 24 hours the number of viable cells was assessed by use of the CyQuant® NF kit (Molecular Probes, Switzerland). The level of apoptosis was determined measuring the caspase −3 and −7 activity (Apo-ONE® Homogeneous Caspase −3/7 Assay, Promega AG, Switzerland). All experiments were performed in quadruplicates with EPC-CM generated from five different EPC donors.

### *In vitro* angiogenesis assay

Aortas isolated from nude rats were cut into 1 mm thick rings and placed individually in a 24-well plate coated with growth factor-reduced Matrigel™ (Becton Dickinson, Germany) [51] and incubated with EPC-CM or control medium. The experiment was performed in quadruplicates for each culture condition. After 5 days of culture, sprout length was calculated digitally using ImageJ.

### *In vivo* experimental design

The *in vivo* experimental design of the study is schematically depicted in Figure 3. Experimental set 1 was designed to investigate the long term effect of the different treatment strategies on hindlimb perfusion and function. Aim of Experimental Set 2 was to evaluate the stimulation and recruitment of host cells involved in the endogenous repair system in response to EPC-CM treatment.

### *In vivo* angiogenesis model

Chronic hindlimb ischemia was induced by unilateral excision of an arterial segment extending from the external iliac to the femoral vessels (artery and vein) in male athymic nude rats (NIH-Foxn1^rnu^, Charles River Laboratory Inc, Sulzfeld, Germany) during 0.2% isoflurane anesthesia. Buprenorphinum (Temgesic®, 0.1 mg/kg, Essex Chemie, Switzerland) was injected subcutaneously at the end of the procedure. Rats were then randomly assigned to 3 treatment groups (n = 8 in each group) for 3 serial intramuscular injections of EPC-CM, EPC or control medium within 7 days (Figure 3). Injections were performed four weeks following arterial occlusion (iDAY1). Each time a total volume of 250 µl EPC-CM or control medium, or 1×10^6^ EPC were injected at 5 sites into the ischemic hindlimb distal to the arterial occlusion site. Three ventral injections were placed in the upper limb in proximity to the adductor and semimembranosus muscles. The remaining 2 injections were administered to the ventral lower limb involving the gastrocnemius and flexor digitorum muscles. In order to achieve maximal experimental uniformity, transplanted EPC and EPC-CM were derived from the same donors and used in parallel experiments. All procedures were approved by the Cantonal Ethics Review Board and conducted in accordance with the institutional policies for animal experiments.

### Laser Doppler blood perfusion imaging

Blood flow in the ischemic and healthy lower hindlimbs was measured weekly until iDAY 35 using a laser Doppler Imager (Moor, Axminster, UK). The animals were placed on a heating pad in order to maintain a constant body temperature during the entire measurement. In each rat the values of two consecutive measurements were averaged and the perfusion was expressed as ratio of values for the ischemic to normal limb [11].

### Assessment of hindlimb function

We adopted a forced swimming test to determine the functional capacity of the ischemic hindlimb after treatment [52]. Animals were placed in a water-filled tank (23°C) to swim. Active strokes per minute of each limb were counted during 3 consecutive periods (0–1 min, 1–2 min, and 2–3 min). Functional muscle activity was calculated as the ratio of number of strokes/min of the ischemic to the healthy hindlimb and compared to non-operated, age-matched healthy rats (n = 5).

### Assessment of muscular viability and immunohistochemistry

All animals were euthanized by use of carbon dioxide five weeks after treatment for analysis of mitochondrial activity and immunohistochemistry. The mitochondrial activity, serving as a surrogate marker of muscular viability, was assessed in the hindlimb muscles by the MTT reduction test. Muscle viability was calculated as the ratio of extracted MTT absorbance values per gram of dry tissue of the ischemic and the healthy contralateral limb [53]. The gastrocnemius muscle of the hindlimb was fixed in 4% formaldehyde for 24 hours and embedded in paraffin. Ten µm thick sections were stained with lectin from *Bandeiraea simplicifolia* (BS-1, Sigma-Aldrich, Germany) or with von Willebrand factor (vWF; AB7356, Chemicon, USA) in order to determine capillary density in the muscle tissue. Vascular mural cells were identified as cells immunoreactive for NG2 (N8912, Sigma-Aldrich, Germany) adjacent to endothelial cells stained with vWF. A mouse monoclonal antibody against rat α-smooth muscle actin (α-SMA, Sigma-Aldrich, Germany) and the LSAB™ - alkaline phosphatase kit (Dako, USA) were used to localize vascular maturation [54]. The number of capillaries, NG2^+^ pericytes and smooth muscle cell-covered vessels was counted in 5 random high power fields (HPF) by use of ImageJ software. Capillary density was expressed as the ratio of capillary numbers per muscle fiber. NG2^+^ pericyte density was expressed as the ratio of cell numbers per capillary. A total of 10 sections from 3 different muscle levels were obtained per animal and analyzed by a blinded investigator.

### Progenitor cells mobilization

To investigate whether the effect of intramuscular applied EPC-CM on tissue regeneration and neovascularization involves the endogenous repair system of bone marrow-derived progenitor cells, we performed experiments according to Experimental Set 2 (Figure 3) to evaluate mobilization, recruitment and homing of progenitor cells [18]. The number of CD34^+^/CD45^−^ progenitor cells was measured in the bone marrow and the peripheral blood of EPC-CM and control media treated animals (n = 5) using flow cytometry 3 days after the last treatment. Immediately following isolation, MNC from BM and PB were processed for FACS analysis. The expression of surface markers CD34 (Santa Cruz, USA) and CD45 (Cedarlane Laboratories, Canada) were measured in a LSR II flow cytometer (Becton Dickinson, USA) using the Cell Quest software (Becton Dickinson, USA). Immunofluorescence staining was used to determine the number of CD34^+^ cells in both adductor and gastrocnemius muscles from ischemic hindlimbs. To compare the recruitment of CD34^+^ cells at different time points, immunofluorescence staining for CD34^+^ cells was also performed in tissue sections from gastrocnemius muscles harvested at iDAY35.

### Statistical analysis

Data are reported as means±SEM unless otherwise stated. Proportions were compared by use of Pearsoǹs X2-Test and Fisher̀s exact test, respectively, applying the Bonferroni correction for repetitive testing. Kruskal-Wallis test and post-hoc comparison with Scheffe's test were used to compare means of continuous variables amongst the different study groups. Statistical significance was inferred at a 2-sided value of P<0.05. Statistics were carried out using SPSS software package (version 16.0; SPSS Inc, Chicago, IL).

The authors had full access to the data and take responsibility for its integrity. All authors have read and agree to the manuscript as written.
